# Infected forearm nonunion treated by bone transport after debridement

**DOI:** 10.1186/1471-2474-14-273

**Published:** 2013-09-22

**Authors:** Tang Liu, Zhenyang Liu, Lin Ling, Xiangsheng Zhang

**Affiliations:** 1 Department of Orthopaedics, the Second Xiangya Hospital, Cental South University, Changsha, 410011 Hunan, PR China; 2 Department of Hemotology, the Second Xiangya Hospital, Cental South University, Changsha 410011, Hunan, PR China; 3 Department of Internal Medical Oncology, The Affiliated Tumor Hospital of Xiangya Medical School of Central South University, Changsha 410011, Hunan, PR China

**Keywords:** Bone transport, Infected nonunion, External fixator, Bone defect

## Abstract

**Background:**

This is a therapeutic study to evaluate the results of the management of forearm infected nonunion using bone transport with external fixators after debridement.

**Methods:**

We have retrospectively reviewed a consecutive series of 21 patients from October 1994 to June 2010 in our institution who were treated for the forearm infected nonunion by bone transport with external fixator after debridement. There were 12 males and 9 females. The mean age of the patients was 27.1 years. Of the initial fractures, nonunion of the radius alone invovled in 7 patients, nonunion of the ulna alone invovled in 12, and nonunion of the radius and ulna invovled in 2. Nineteen limbs (85.7%) were in active infected state with sinus and drainage. The mean amount of bone defect was 3.1 cm (range 1.8-4.6 cm) as measured on plain radiographs.

**Results:**

The mean follow-up was 77.5 months. All patients achieved bony union and were satisfied with the functional and cosmetic outcome. All the infection had been controlled. The mean external fixation index was 42.5 day/cm. The average time for wound healing was 42 days. The mean length gained was 3.5 cm (2.1-5.3 cm).

**Conclusions:**

The technique of bone transport after debridement is a safe, effective, and minimally invasive treatment for forearm infected nonunion.

## Background

Infected forearm nonunion is an infrequent complication of diaphyseal fracture of the forearm, which is a challenge for a orthopaedic surgeon [1,2]. The problem is complex due to the presence of bone necrosis, segmental bone loss, sinus tract formation, fracture instability, and scar adhesion of the soft tissues [1,2]. Reviewing the literature of this subject reveals that despite the use of various techniques for treating forearm nonunions, the results are not completely satisfying and there is still debate regarding which type of technique to use [1-9]. In this study, we evaluated the results of 21 forearm infected nonunions via bone transport with a unilateral external fixator after debridement.

## Methods

Twenty-one consecutive patients with post-traumatic forearm infected nonunions were treated from October 1994 to June 2010 in the 2^nd^ Xiangya Hospital. Bone transport with an external fixator was used in all patients. This study was approved by the 2^nd^ Xiangya Hospital committee for clinical research and informed consent was obtained from the patients participating in the study(2000-S02). The patients provided written informed consent for the publication of individual clinical details and accompanying images.

There were 12 males and 9 females (see Table 1). The mean age of the patients was 27.1 years (range 15-56 years). The injury mechanisms included: 5 fallings, 13 traffic accidents, and 3 crush injuries. The initial fracture involved the radius alone in 4 forearms, the ulna alone in 2 forearms, and the radius and ulna in 15 forearms, in which one of the bones had subsequently been healed in 13 patients. Five patients sustained open fractures. The initial treatment consisted of fixation with a plate and screws in 21 bones, an intramedullary rod in 7, external fixation in 4, and cast immobilization in 4. Of the initial fractures, nonunion of the radius alone involved in 7 patients, nonunion of the ulna alone involved in 12, and nonunion of the radius and ulna involved in 2. The average interval between their initial treatment and administration to our department was 15.6 months (range 10-68 months).

**Table 1:** Details of the patients

**Case**	**Age**	**Nonunion site**	**Bone defect**	**Wrist flexion/extension (°)**		**Forearm pronation/supination (°)**		**Length gained**	**External fixation index**	**Wound healing time**
	**(years)/sex**		**(cm)**	**Pre-op**	**Follow-up**	**Pre-op**	**Follow-up**	**(cm)**	**(days/cm)**	**(days)**
1	18/M	Ulna, middle	2.1	35–0–40	50–0–40	55–0–60	65–0–80	2.5	42.1	43
2	35/F	Radius, proximal	3.4	20–0–25	55–0–40	40–0–35	60–0–65	3.5	46.5	47
3	25/M	Ulna, distal	1.8	25–0–40	65–0–50	45–0–50	60–0–75	2.0	39.6	39
4	56/F	Radius, proximal	2.9	30–0–25	50–0–45	35–0–40	65–0–80	3.0	51.6	54
5	15/M	Ulna, distal	3.5	60–0–55	60–0–55	55–0–40	80–0–85	3.5	37.9	31
6	37/M	Ulna, distal	4.6	50–0–50	50–0–50	60–0–45	75–0–70	4.8	47.5	57
7	43/F	Both, distal	2.4	10–0–15	45–0–30	25–0–30	50–0–45	2.5	50.5	42
8	51/F	Radius, distal	4.2	25–0–30	55–0–40	40–0–55	60–0–75	4.5	53.2	53
9	40/M	Ulna, proximal	4.0	30–0–30	50–0–60	55–0–40	85–0–80	4.0	38.0	36
10	24/F	Radius, middle	3.7	20–0–25	60–0–55	30–0–45	65–0–60	4.0	45.6	48
11	27/M	Ulna, distal	2.9	30–0–45	40–0–60	45–0–60	75–0–80	3.0	43.5	35
12	32/M	Ulna, proximal	3.6	35–0–35	50–0–50	30–0–45	60–0–75	3.8	47.3	52
13	38/M	Radius, proximal	2.3	50–0–60	50–0–60	45–0–50	75–0–70	2.5	39.5	37
14	45/F	Ulna, distal	3.4	60–0–60	60–0–60	75–0–60	75–0–80	3.5	41.5	41
15	31/F	Ulna, distal	4.1	30–0–25	50–0–40	20–0–35	85–0–75	4.0	47.5	53
16	26/M	Radius, distal	3.8	25–0–30	35–0–50	40–0–25	60–0–50	4.0	51.2	36
17	17/F	Ulna, proximal	2.6	40–0–45	60–0–60	50–0–40	75–0–80	3.0	43.5	38
18	36/M	Ulna, distal	3.2	30–0–45	40–0–50	40–0–45	65–0–60	3.5	53.5	46
19	29/F	Both, distal	2.7	20–0–15	40–0–60	40–0–35	65–0–70	3.0	45.7	43
20	21/M	Ulna, distal	4.5	30–0–45	60–0–60	55–0–30	70–0–80	4.5	39.5	35
21	38/M	Ulna, proximal	3.8	40–0–45	40–0–45	30–0–40	65–0–70	4.0	43.0	42

In the present study group, all the patients had a segment bone defect, and an average of 3.2 surgical procedures (range 1-8 procedures) before presenting to our institution. 19 limbs (85.7%) were in active infected state with sinus and drainage. The rest were quiescent. There were plates and screws left in 20 limbs. The mean amount of bone defect was 3.1 cm (range 1.8-4.6 cm) as measured on plain radiographs.

### Surgical technique

Pre-operative radiographs were taken in the sagittal and coronal planes to assess the length inequality between the radius and ulna and to plan the application of the unilateral external fixator. Then management of infected nonunion was performed by bone transport after debridement in the same surgical process.

#### Step 1: Eradication of infection and restoration of the tissue defects

For the infected limbs, particularly those that still had sinus and active discharge, hardware removal and radical resection of dead bone with debridement of the infected scarred soft tissue were performed, and representative tissue cultures samples, including the sinus tracts for all dead bones were obtained. Cortical bleeding, described as the so-called paprika sign, was accepted as an indication of vital tissue [10]. Wounds were rinsed with pulsed irrigation system and sterilized with iodine complex. Then, all instruments, operating gowns, gloves and sterile towels were replaced. In all cases, the medullary canal was opened using a drill to ensure bone bleeding. The dead space was filled with custom-made iodoform gauze.

#### Step 2: Bone transport with external fixation

After complete debridement, all patients were performed bone transport using external fixators to restore bone at bone defect. In those cases presenting nonunion in both radius and ulna, fixation of the ulna was performed first to restore bone length and alignment. Under image intensifier control, one or two pins (diameter 3.0 mm) were inserted 2 cm to 3 cm above and below the pre-selected osteotomy site. Each set of pins should be in the same plane perpendicular to the anatomical axis of the ulnas or radius. Further pins were inserted into the distal ulna or radius segment if necessary. A dorsal incision was used in the forearm for osteotomy. The periosteum was incised longitudinally and reflected medially and laterally. Under direct vision, a series of 1.5 mm unicortical drill holes were made in the anterior two-thirds of the circumference of the bones and connected with an osteotome. The ulnas or radius was flexed to crack the posterior cortex, completing the corticotomy. The periosteum was sutured and the wound closed with a drain and a monolateral external fixator applied.

### Post-operative protocol

Antibiotic therapy was based on the results of Gram’s stain and culture of organisms from tissue specimens or pus obtained at surgery before beginning antibiotic therapy. After surgery patients were treated with 6 weeks of intravenous antibiotics selected by an infectious disease specialist. Thereafter, patients received appropriate oral antibiotics for 3 months or until the end of surgical treatment. Physiotherapy was started on the second day after operation which included range of movement exercises for the fingers and elbow as well as the use of dynamic slings for finger extension. The patients were encouraged to train the affected limbs through pushing wall as stress simulation. The used idoform gauze was removed when the wound was ready to be cleaned and repacked, which was usually done daily. The gauze should be easy to remove, and it should be disposed off properly once it is removed. The wound was repacked using an idoform strip 1 to 3 cm shorter than the initial piece.

Distraction was begun between five and seven days after the operation at a rate of 0.25 mm per nine hours [1,2]. The rate was adjusted according to the discomfort and swelling of the hand and the quality of the regenerated bone. During the lengthening, all the patients were followed up every two weeks. Anteroposterior and lateral radiographs of the forearm were taken to monitor bone regeneration, measure the bone loss and adjust the rate of lengthening. Once the required length was achieved, distraction was discontinued. The unilateral external fixator was removed when sufficient consolidation was obtained, i.e. when at least three of four cortices were observed to be united on anteroposterior and lateral radiographs. After removal of the external fixator, a cast was applied for three weeks.

## Results

**Figure 1:**
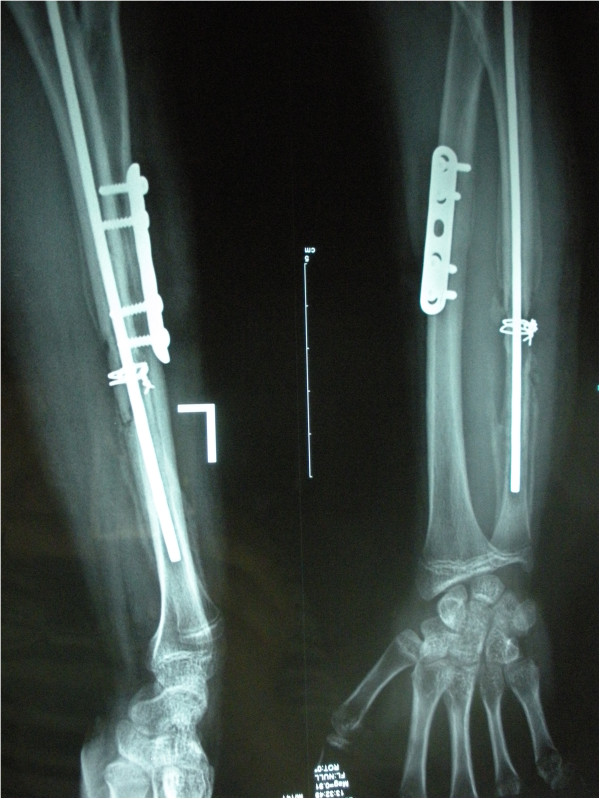
Radiograph of a 15-year-old man (case 5) who sustained comminuted fractures of ulna and radius had an infected ulna nonunion.

**Figure 2:**
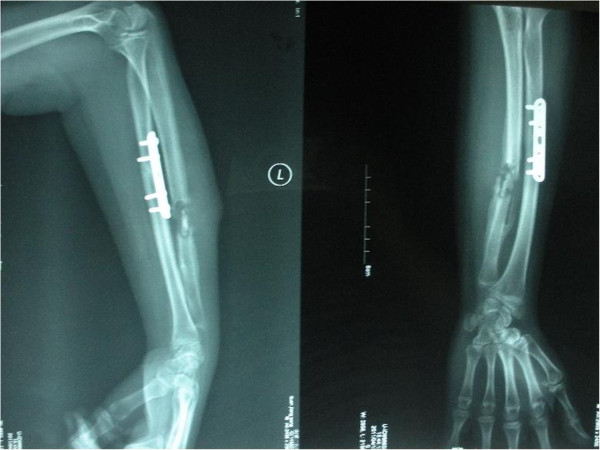
**It shows dead bone in the docking site after removal of the ulna internal fixation.** The ulna defect was 3.5 cm.

**Figure 3:**
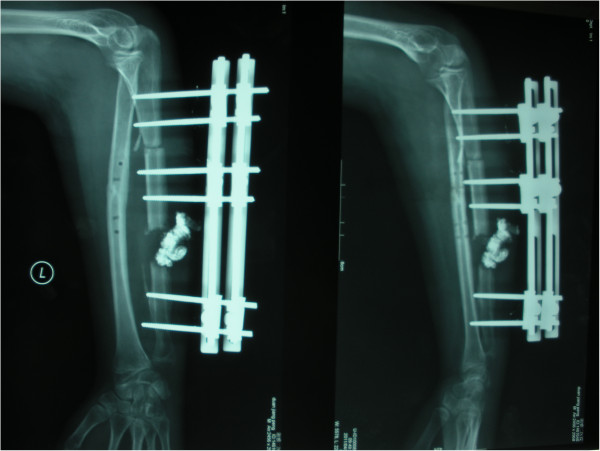
**3 days after operation.** It shows removal of the internal fixations, eradication of the necrotic bone and tissues and application of the three-segment unilateral external fxator. The wounds were packed with iodoform gauze additionally.

**Figure 4:**
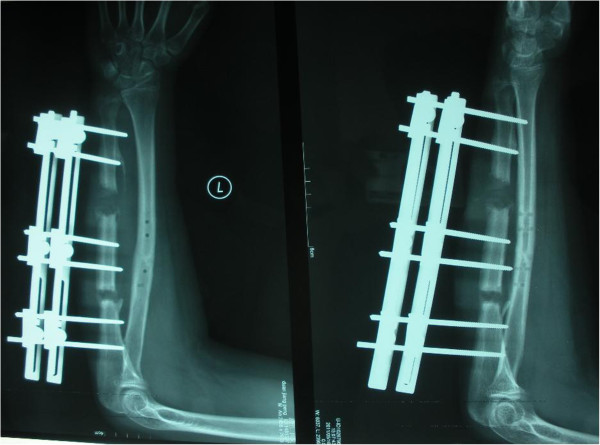
20 days after compression-distraction osteogenesis with the external fIxator.

**Figure 5:**
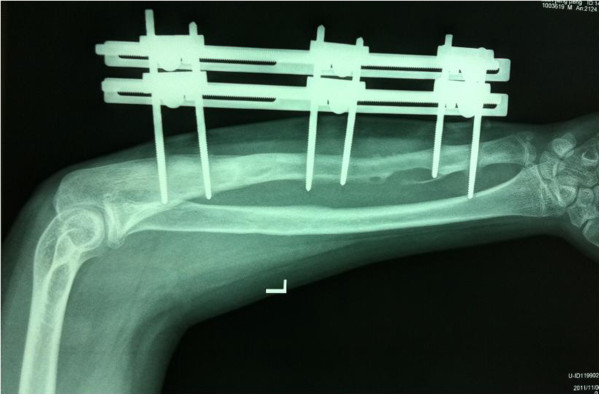
4.5 months after the operation, the callus regenerated well to prepare to remove external fixator.

The mean follow-up was 77.5 months (range 21–136 months). All the patients had bony union and the infection had been controlled (see Figures 1, 2, 3, 4 and 5). The mean external fixation index was 42.5 day/cm (range 37.9-51.6 day/cm). The average wound healing time was 42 days (range 31-57 days). No patient required flap coverage. The mean length gained was 3.5 cm (2.1-5.3 cm). Wrist mobility was painless and improved considerably in all patients. Flexion improved from a mean of 33.1° (10° to 60°) pre-operatively to 50.7° (35° to 60°) at the last follow-up, extension from 37.4° (15° to 60°) to 51.0° (30° to 60°), pronation from 43.3° (25° to 75°) to 68.3° (50° to 85°), and supination from 43.1° (25° to 60°) pre-operatively to 70.7° (45° to 80°) at final follow-up. Elbow and finger mobility returned to their pre-operative state, and grip strength was markedly improved (see Table 1).

### Complications

Pain was the most common complaint during the distraction period, which was observed particularly in patients requiring lengthening in excess of 4.0 cm, and was relieved consistently by oral analgesics. Twelve patients (57.1%) had a pin-track infection. Ten of these had local inflammation, which had been settled with pin care and oral antibiotics, and two had a purulent drainage, which had been settled with intravenous antibiotics. Loosening of a pin during distraction occurred in six limbs (28.6%). Five of them were treated by reapplied pins and one was treated by reapplication of the external fixator for further lengthening. Cancellous bone grafting for delayed union at the docking site was required in four patients (19.0%, case 4, case 8, case 16 and case 18), who proceeded to union. Poor regenerated bone formation occured in two limbs (9.5%, case 7 and case 15), which also required bone grafting for union at last. Three patients (14.3%, case 6, case 10 and case 12)had failures in the form of recurrent drainage. They underwent repeated debridements, and the aeschynomenous antibiotic solution was used to make continuous aspiration lavage drainage with two tubulars for three weeks. Subsequently, in these three patients, the infection was eradicated and the nonunion was healed, resulting in good function and a good radiographic outcome.

There were no refractures or neurovascular complications.

## Discussion

The present study showed good functional and cosmetic outcomes following bone transport with external fixators after debridement for forearm infected nonunion. All patients achieved bony union and the infection was controlled.

Reviewing the literatures, most reports on the treatment of infected nonunions refer to the lower extremity, particularly the tibia [10-13]. Infected nonunion in the upper extremity is a rare event, especially in the forearm [1]. Such patients usually have had numerous previous surgical interventions, resulting in bone defects and soft tissue compromise [1,2]. The most common treatment for bone defect is bone grafting. Prasarn et al [1] reported 15 patients with an infected nonunion of the diaphysis of the radius or ulna a protocol that combines aggressive surgical debridements as necessary, definitive fixation after 7–14 days, tricortical iliac crest bone grafting for segmental defects, leaving wounds open to heal by secondary intention, 6 weeks of culture-specific intravenous antibiotics, and early active range of motion exercises. At follow-up 2–15 years, all patients had united and resolved their infections. Ring et al [2] have reported on a large series of nonunions of diaphyseal forearm fractures. Their retrospective review consisted of a total of 35 patients, of which 11 had deep infections. They also treated the segmental defects with autologous bone graft. But they did not provide a detailed description of their treatment protocol for the infected patient subset, and never commented on whether the infection was completely eradicated following treatment. Though autologous cancellous bone graft is an effective way to treat small defects, it is not enough to fill massive defect. In addition, the healthy tissue may be sacrificed and the grafted bone may be absorbed [11]. Vascular bone grafting has been proven useful in overcoming massive bone defects. This type of graft can be transferred as an osteoseptocutaneous flap; thereby reconstructing the soft tissues as well as the bone. However, healing and remodelling of bone graft are lengthy; osteopenia and joint stiffness due to prolonged remodeling may occur. In addition, refracture and host–graft junction healing problems also are common complications with this type of grafting technique. Cierny et al [14] compared the results of treating segmental tibial defects using Ilizarov bone transport and massive autologous bone graft, and the results were in favor of the Ilizarov method. Cierny et al’s study [14] stated that Ilizarov methods had lower complication rates when comparing with massive cancellous grafts. In addtion, they confirmed that Ilizarov reconstructions averaged fewer hours in the operating theater, fewer days in the hospital, fewer months’ disability, and lower cost for applications [14].

In the present study, all the patients were treated by bone transport. To avoid delayed bone-healing, complete cure of the infection is the main stay of treatment in infected nonunions. The most important principle in eradicating osteomyelitis is thorough and adequate debridement until live and bleeding bone is reached. In the author’s opinion, successful treatment of infected nonunion often combines radical debridement of the septic bone and soft tissue in addition to application of stable fixation to enhance soft tissue healing and bone union. So, the radical removal of the necrotic and infected parts of both bone and soft tissues represents the most important element for the success of treatment by compression-distraction technique in severe infected nonunion of the ulna or radius. Loose and sequestrated bone should be also removed. In the present study, the infection had been controlled in all the patients.

It is a critical problem that whether the lengthened area will be infected secondarily when the bone is being debrided and lengthened in patients with infected nonunion and bone defect. To our knowledge, there is no report on lengthened area infection in infected nonunion patients after simultaneously bone transport and debridement. In the present study, there is also no lengthened area infection. The authors adopted a remote osteotomy to start the distraction and sufficient drainage in infective site. The osteotomy was performed in the healthy bone far from the infected bone to ensure good vascularity for consolidation.

Bone transport in the forearm may cause damage to the neurovascular structures particularly in patients with scarring from osteomyelitis and previous failed operations [1,2]. There was no evidence of neurovascular injury in any of our 21 patients. The healing index and overall rate of complications were similar to those of previous reports in which a circular fixator had been used [15]. A monolateral external fixation system can provide stable fixation at the site of the osteotomy without the use of tensioned transfixion wires. Some authors consider that there is less risk of neurovascular damage when using a monolateral external fixator [16]. However, delayed union at the docking site was observed in four patients and poor regenerated bone formation occurred in two limbs. In these cases, union was achieved following bone grafting. In our opinion, one of the reasons for poor regenerated bone formation is that, previous surgical intervention in which an osteotomy have to be performed through an insufficiently vascularized area. We believe that, for patients who have had previous surgery, multiple drill-hole osteotomies made through fresh bone are preferable as they preserve the periosteum and surrounding musculature. During rehabilitation, patients should be suggested to train the affected limbs through pushing wall as axial stress simulation.

The weaknesses of our study are the absence of a control group and our small number of patients. Nevertheless, the present study described a new and successful alternative technique for the treatment of the challenging problem of infected forearm nonunions.

## Conclusions

The technique of bone transport with the external fixator after debridement is a safe, effective, and minimally invasive management to treat the forearm infected nonunion.

## Competing interests

The authors declare that they have no competing interests.

## Authors’ contribution

TL accountable for the execution of the research, the integrity and analysis of the data, and the writing of the manuscript. ZL accountable for the integrity and analysis of the data, and the writing of the manuscript. LL accountable for the the conception and execution of the research. XZ accountable for the conception and execution of the research. All authors read and approved the final manuscript.

## Authors’ information

Tang Liu and Zhenyang Liu are co-first authors.

## Pre-publication history

The pre-publication history for this paper can be accessed here:

http://www.biomedcentral.com/1471-2474/14/273/prepub
